# Relating individual motion sickness levels to subjective discomfort ratings

**DOI:** 10.1007/s00221-022-06334-6

**Published:** 2022-02-22

**Authors:** Ksander N. de Winkel, Tuğrul Irmak, Varun Kotian, Daan M. Pool, Riender Happee

**Affiliations:** 1 Delft University of Technology, Mekelweg 2 2628 CD, Delft, South Holland Netherlands; 2 Delft University of Technology, Kluyverweg 1 2629 HS, Delft, South Holland Netherlands

**Keywords:** Motion sickness, Comfort, Discomfort, MIsery SCale

## Abstract

High levels of vehicle automation are expected to increase the risk of motion sickness, which is a major detriment to driving comfort. The exact relation between motion sickness and discomfort is a matter of debate, with recent studies suggesting a relief of discomfort at the onset of nausea. In this study, we investigate whether discomfort increases monotonously with motion sickness and how the relation can best be characterized in a semantic experiment (Experiment 1) and a motion sickness experiment (Experiment 2). In Experiment 1, 15 participants performed pairwise comparisons on the subjective discomfort associated with each item on the popular MIsery SCale (MISC) of motion sickness. In Experiment 2, 17 participants rated motion sickness using the MISC during exposures to four sustained motion stimuli, and provided (1) numerical magnitude estimates of the discomfort experienced for each level of the MISC, and (2) verbal magnitude estimates with seven qualifiers, ranging between feeling ‘excellent’ and ‘terrible’. The data of Experiment 1 show that the items of the MISC are ranked in order of appearance, with the exception of 5 (‘severe dizziness, warmth, headache, stomach awareness, and sweating’) and 6 (‘slight nausea’), which are ranked in opposite order. However, in Experiment 2, we find that discomfort associated with each level of the MISC, as it was used to express motion sickness during exposure to a sickening stimulus, increases monotonously; following a power law with an exponent of 1.206. While the results of Experiment 1 replicate the non-linearity found in recent studies, the results of Experiment 2 suggest that the non-linearity is due to the semantic nature of Experiment 1, and that there is a positive monotonous relation between MISC and discomfort in practice. These results support the suitability of MISC to assess motion sickness.

## Introduction

It is expected that the introduction of automated vehicles will provide major benefits in terms of safety, utility, and comfort to passengers as well as having beneficial effects on the environment through increased driving efficiency. However, a major hindrance to widespread adoption of the technology, thus negating the projected benefits, is its potential to increase motion sickness (Diels and Bos 2016). To prevent and mitigate this issue as much as possible, it is necessary to understand the aetiology of motion sickness and determine its relation to discomfort. This in turn requires reliable tools to measure these states experimentally.

Motion sickness may be quantified using physiological measures or subjective rating scales. Physiological measures, such as Electro-GastroGraphy (EGG; e.g., Gruden et al. 2021) or the Galvanic Skin Response (GSR; e.g., McClure et al. 1971; Cowings et al. 1986; Irmak et al. 2020) show correlations with sickness ratings, but these correlations are not perfect and the measurements tend to have limited specificity. For instance, skin conductance (GSR) varies due to sweating, which depends on motion sickness but also on ambient factors such as temperature. Due to such issues, subjective rating scales are currently the preferred method to quantify motion sickness.

The MIsery SCale [MISC, or, more aptly: “Motion Illness Symptoms Classification scale” (Reuten et al. 2021a)] is a rating scale that is commonly used to express the level of motion sickness a person experiences. The scale spans 11 discrete points, ranging between 0, which corresponds to ‘no problems’, to 10 for ‘vomiting’ (see Table 1). In contrast to a number of alternative rating scales such as the Well-Being scale (Reason and Graybiel 1969) or the Fast Motion Sickness (FMS) scale (Keshavarz and Hecht 2011), each level is anchored to specific symptoms, ordered on the basis of a consensus on the progression of motion sickness symptoms over time and increasing levels of discomfort (Wertheim et al. 1998; Bos et al. 2005). The ratings can be given verbally and instantaneously. As such, administering the MISC is minimally invasive among any other experimental procedures, and it has proven to be a convenient and popular tool to monitor the progression of motion sickness symptoms over time (e.g., Irmak et al. 2020, 2021). Early versions of the MISC were developed to rate sickness experienced on ships (Wertheim et al. 1998), and were based on the notion that although the severity of symptoms varies between individuals, symptoms such as dizziness, hot or cold flushes, headache, stomach awareness, and sweating precede nausea and vomiting. The version of the scale that has been broadly adopted was designed by Bos et al. (2005). The authors performed a validation study where a sample of 24 participants were exposed to 30 min periods of simulated ship motion on a moving base simulator, under three different visual conditions. After the simulator runs, the participants ranked (1) the order of appearance and (2) the severity of 27 different symptoms. From the responses on these tasks, group-level order and severity indices were calculated. To validate the MISC, a linear regression analysis was subsequently performed to predict the MISC; using 25 observed symptom severities as regressors (retching and vomiting were excluded, as these did not occur). Although this procedure yielded predictions of the MISC that corresponded well with observed data, there are some potential issues with the methodology.

First, the MISC was predicted using 25 regressors, whereas the MISC itself features five pooled categories that cover only a subset of symptoms. It may be questioned whether symptoms that are not covered by the MISC should be used for its prediction, since any symptom indicative of motion sickness arguably contributes to the experience thereof. Moreover, the regression procedure yielded negative coefficients with considerable weights for nine regressors, including sweating and pallor. This suggests that increased sweating and pallor are related with less sickness, which is unlikely, and which thus may reflect a modeling artefact.

Second, the rationale for pooling symptoms other than nausea was that these should precede nausea in a particular order. However, the ordering of symptoms in the scale does not correspond exactly to participant reports. Notably, sweating was included in the scale as a symptom preceding nausea, along with dizziness, warmth, headache, and stomach awareness, whereas participants appeared to consistently rank sweating as a symptom which occurred after nausea was first experienced (Bos et al. 2005).

Third, using a single scale, it is implicitly assumed that there exists a single latent motion sickness variable that individuals query to provide responses on the MISC. This assumption may be questioned, as factor analyses performed on motion sickness questionnaire data typically identify at least three factors. For instance, the Simulator Sickness Questionnaire (SSQ) identifies Nausea, Disorientation, and Oculomotor factors (Kennedy et al. 1993), thus differentiating between symptoms that appear to be orchestrated at different levels of the nervous system (i.e., peripheral, autonomous, or central). Consequently, it may be that the MISC confounds neurologically distinct clusters of motion sickness responses that may each have their own time course.

The issues described above may lead to a number of problems with the analysis and interpretation of MISC data. Specifically, these problems can be classified as issues of *ordinality* and of *linearity*. *Ordinality* refers to the ordering of items on the scale. The fact that motion sickness may express with more symptoms than are covered in the scale suggests that it may be incomplete and therefore not reflect the overall sensation of motion sickness. If the ordering of symptoms does not accurately reflect their actual order of appearance, or if the time courses of distinct neurological clusters of symptoms are not accurately reflected in the ordering of the MISC, then a strict application of the scale may (erroneously) suggest improvements of well-being in the form of lower scores (i.e., reduced discomfort) for increasing overall levels of motion sickness. *Linearity* relates to uniformity of the distance between points on the latent sickness variable queried to produce MISC scores. Although subsequent scores on any ordinal scale should increase monotonously with the latent variable, the distance between successive scores is unknown (Stevens 1946). These distances may not be constant, and may differ between individuals. General methods exist to deal with this, such as ordinal regression (Scott Long 1997). However, MISC data are typically analysed using linear regression models or other techniques that make the assumption that the data form an interval scale (e.g., Bos et al. 2005; Irmak et al. 2021). Violation of this assumption may affect the validity of conclusions that follow from such analyses (Winship and Mare 1984). For instance, if a predictive mathematical model of motion sickness is fitted to MISC data obtained for motions of a certain amplitude, the model may not yield accurate predictions for other amplitudes if it does not account for such non-linearities; possibly leading to spurious conclusions on the ability of a model to describe motion sickness. As an illustration of these issues, (Reuten et al. 2020, 2021a, b) report a striking discontinuity in the relation between MISC scores and subjective discomfort levels (there referred to as “unpleasantness”) associated with each score, suggesting that a MISC score of 6 is associated with less discomfort than a MISC score of 5.

The aim of the present study was to evaluate whether the potential issues with ordinality and linearity affect the validity of using the MISC as a quantitative measure of sickness level, and to relate the progression of motion sickness symptomatology expressed using the MISC to the experience of discomfort. Specifically, we seek to answer the following research questions:

1. Does discomfort increase monotonously with MISC score?
2. How can the relation between MISC and discomfort be characterized?

We hypothesize that the MISC is used to express an overall sensation of motion sickness and that discomfort increases monotonously with MISC score. In addition, we hypothesize that the relation between MISC and discomfort can be characterized using a Stevens power law (Stevens 1957).

## Methods

To address our research questions, we performed two experiments. The first experiment is a replication of an experiment performed by Reuten et al. (2020, 2021a, b). Here, we asked a sample of individuals to discriminate between the discomfort levels they subjectively associated with each level of the MISC in a forced choice paradigm from behind their desk; a setting which does not provoke motion sickness. We will refer to this experiment as the *Semantic experiment*. In the second experiment, participants were presented with sustained oscillatory motions at four different amplitudes. At the conclusion of each experimental trial, participants provided Magnitude Estimates (ME) for the level of discomfort they associated with each MISC score they reported during the experiment. At the end of the experiment, they provided ME for a series of verbal qualifiers as a means to interpret the discomfort associated with MISC scores. We will refer to this experiment as the *Motion sickness experiment*. An in-depth analysis of the amplitude dynamics of motion sickness will be reported elsewhere (Irmak et al., in preparation). In the following, we describe the experiments in detail separately.

### Ethics statement

All participants provided their informed consent prior to participation. the experimental protocols were approved by the ethical committee of the Human Research Ethics Committee in TU Delft, Delft, The Netherlands under applications 1834 (Experiment 1) and 1425 (Experiment 2).

### Experiment 1: semantic experiment

#### Participants

Fifteen participants took part in Experiment 1. Participants were all male and between 20 and 38 years old (mean: 25.8, sd: 5.4). Participants were either staff or student at Delft University of Technology, and four were familiar with the MISC. Participants were not reimbursed for participation. The sample may be considered a convenience sample. Due to the COVID-19 pandemic, it has been particularly difficult to recruit participants. Given that effects of demographics such as gender, age, and anxiety on motion sickness are known, and since we do not have any reason to assume that there are differential effects relating to these characteristics for the present research questions, we do not believe that the relative homogeneity of the sample is problematic.

#### Task & stimuli

Participants performed pairwise comparisons for each possible combination of the items of the MISC, answering the question ‘Which is worse?’. The levels of the MISC are given in Table 1 (associated scores were not shown to participants). There were 55 possible unique combinations. The order of comparisons was randomized. Completing the experiment took approximately 5 min.

**Table 1:** MIsery SCale (MISC) and associated symptoms

MISC score	Symtoms
0	No problems
1	Uneasiness
2	Vague dizziness, warmth, headache, stomach awareness, sweating
3	Slight dizziness, warmth, headache, stomach awareness, sweating
4	Fair dizziness, warmth, headache, stomach awareness, sweating
5	Severe dizziness, warmth, headache, stomach awareness, sweating
6	Slight nausea
7	Fair nausea
8	Severe nausea
9	(Near) retching
10	Vomiting

#### Data analysis

For each individual, the number of times *C* a symptom was rated as worst was calculated and normalized using the equation $\text {Pr}(\text {worst}) = (C-C_\text {min})/(C_\text {max}-C_\text {min})$ (Reuten et al. 2020, 2021a, b). We compared the absolute values of the means for each level.

### Experiment 2: motion sickness experiment

#### Participants

Data for 17 participants were collected in Experiment 2. This was an independent sample from those of Experiment 1. From 16 participants, Verbal Qualifier data were obtained. These data were used for the analysis presented here. All but two participants were male. They were between 22 and 32 years old (mean: 25.4 sd: 2.6). Participants were either staff or student at Delft University of Technology, and two were familiar with the MISC. Participants were reimbursed with a €10 voucher for participation. As in Experiment 1, this sample could be considered a convenience sample. However, given the arguments presented there, we do not believe that the relative homogeneity of the sample is problematic.

#### Task & stimuli

In the experiment, participants were exposed to prolonged periods (60 min or until MISC 6 was reached, followed by a 10 min break, and then another 30 min of motion or until MISC 6; i.e., a maximum of 90 min of motion with a 10 min break after 60 min) of fore–aft oscillation at a fixed frequency of 0.3 Hz in the SIMONA Research Simulator at the TU Delft Aerospace faculty. The amplitude of the oscillations was varied in four separate sessions, each presenting motion at one of the following levels ${1, 1.5, 2, 2.5}\text { m/s}^2$. Participants were seated in the simulator cabin and performed the experiment in total darkness. Each participant underwent each level of acceleration once. There was at least a 1-week interval between sessions to prevent habituation, and the order of amplitudes was randomized. In each session, participants rated their level of sickness using the MISC on 30 s intervals.

At the end of each session, they were asked to provide ME of the subjective level of discomfort corresponding to each level of sickness as it was experienced during the session. The MISC items were listed on a single sheet of paper as numbered descriptions; similar to Table 1. Given that participants used the MISC throughout the experiment, participants are likely to know or recognize the order of individual items. Therefore, we chose not to apply a randomization of item order on the response sheet, nor to omit the numbering. Participants were instructed to start at MISC ‘4’ and to attribute this an ME of ‘100’, after which they could rate the remaining items in any preferred order. No additional instructions were given on limitations of the range of numbers. The value of ‘100’ was chosen arbitrarily, so there is no explicit reason this value should be chosen. However, if people were to use a linear scale for their responses, a value of ‘100’ for MISC 4 would provide an overall range of 0–250 for MISC 0–10, which provides ample room to choose responses precisely (i.e., 25 points per MISC score). At the end of each experimental session, participants were furthermore asked to attribute ME to 7 verbal qualifiers, using the same scaling they used to rate sickness during the session. These verbal qualifiers were: ‘Terrible’, ‘Very bad’, ‘Bad’, ‘So–so’, ‘Good’, ‘Very good’, and ‘Excellent’. The verbal qualifiers were presented in random order. The purpose of collecting the verbal qualifier data was to allow an interpretation of the subjective level of discomfort associated with the numerical ME, and thereby of the discomfort associated with motion sickness also between individuals (Venrooij et al. 2015). Participants were told there were no limitations on the scaling of these associations, and that negative values were allowed.

#### Data analysis

**Fig. 1:**
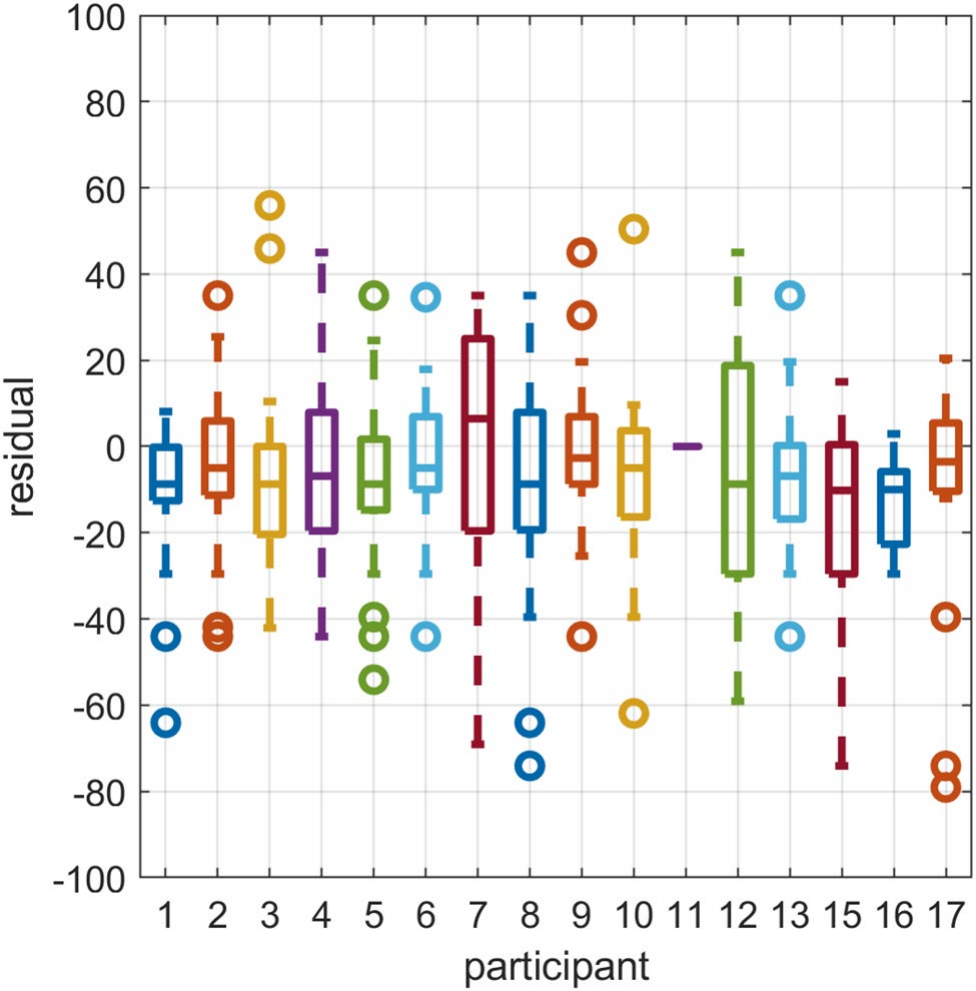
Residuals of the power law model fitted to joint data of all participants in Experiment 2, with each box showing the residuals for a given participant. Data for participant 11 are set to zero in this graph, because this individual used a much larger response scale than the others (up to 1000, compared to a typical value of 200), resulting in residuals that dwarfed those of the other participants in the figure. Note how the residuals are not centred at zero, but are either above or below for different individuals, indicating that individual differences exist that cannot be accounted for by a joint fixed-effects model

ME responses obtained in the Experiment 2 ranged between 0 and 1000, with a median value of 90. The 5th and 95th percentiles were 10 and 200, respectively. Values of responses were chosen by the participants, such that they found them appropriate to express the discomfort experienced for each MISC score, relative to MISC 4, which was to be attributed a value of 100. As initial benchmark models, we fitted a linear model of the form: $\text {ME} = a\times \text {MISC}$, and a power law model of the form $\text {ME} = a\times \text {MISC}^b$ to the joint data of all sessions for all individuals. An intercept was not included, which corresponds to the assumption that there is an absolute zero for MISC corresponding to ‘no problems’ and no discomfort. We then inspected the residuals of this common model for the data of each individual (Fig. 1). This inspection showed that residuals tended to be centred either above or below zero, illustrating that there are individual differences that are not well accounted for by a fixed-effects model. Therefore, we performed further analyses using (1) individually fitted linear and power law models, and (2) a non-linear mixed-effects model, which allows us to account for individual variability of the power law model coefficients (*a*, *b*). For the latter analysis, we followed the procedure outlined in the documentation of the MATLAB Statistics and Machine-learning toolbox on ‘Mixed-Effects Models Using nlmefit and nlmefitsa’. Model comparisons were based on the Akaike Information Criterion (*AIC*, Akaike 1974) and Bayesian Information Criterion (*BIC*, Schwarz 1978). These are model fit indices based on the model likelihood, and include a penalty based on the number of parameters, and in the case of the *BIC* also the number of observations. Lower scores are better. For these fits, the data of all sessions were combined, but session was not included as a predictor in the model. More elaborate models, which allowed for variation of the model exponent *b* between sessions or between amplitudes, did not show a consistent improvement in fit over the simpler model, nor were there significant differences in the value of *b* between sessions. This indicates that participant responses were consistent between sessions. A summary of these additional analyses is given in Appendix 1 of the supplementary material.

**Fig. 2:**
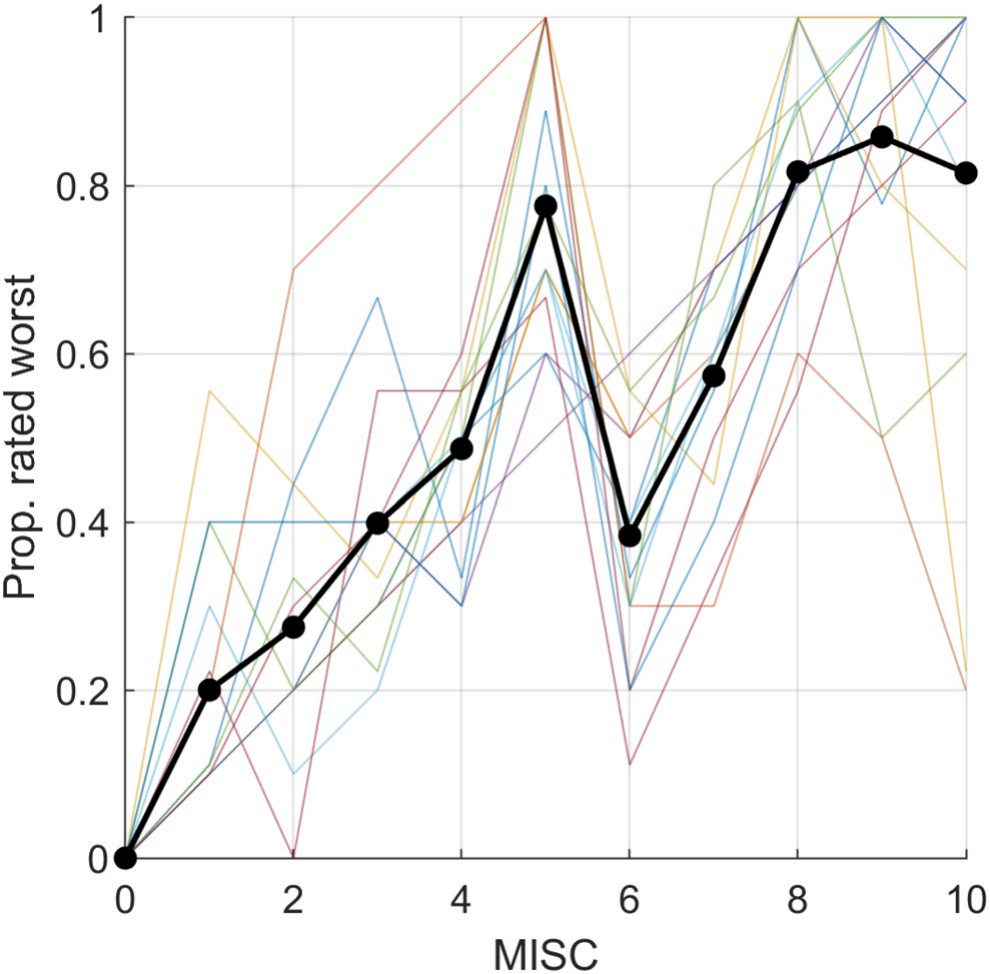
MISC scores versus the proportion of times an individual (thin lines) rated the particular symptom as the worst in comparison with other symptoms as obtained in Experiment 1. The thick black line represents the average over the 15 participants. Note the distinct drop between MISC scores of 5 and 6, which corresponds to observations reported in Reuten et al. (2020, 2021a, b). This drop implies that people find severe dizziness, warmth, headache, stomach awareness, and/or sweating considerably more uncomfortable than slight and even fair nausea

All data generated or analysed during this study are available as supplementary information files.

## Results

### Experiment 1: semantic experiment

The data obtained in Experiment 1 are shown in Fig. 2. The data show that the subjective severity of particular symptoms tends to increase with MISC score, but with a distinct drop between scores of 5 and 6. There is also a notable drop at the end of the scale (“vomiting”). As per anecdotal data obtained during debriefing, this was due to responses of a few individuals who associated vomiting with a relief of motion sickness symptoms.

### Experiment 2: motion sickness experiment

**Fig. 3:**
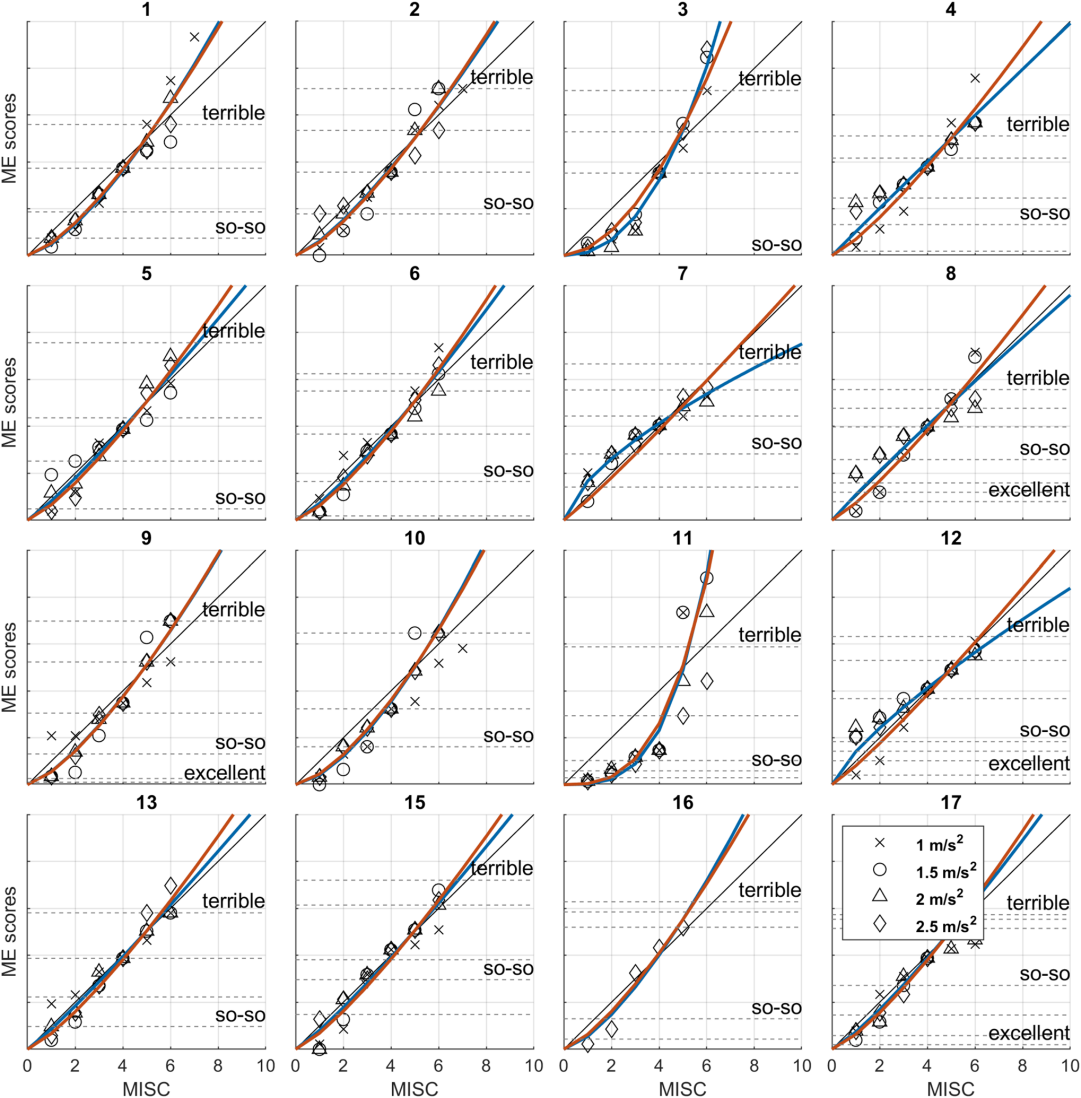
MISC scores and associated Magnitude Estimates (ME) for each individual participant. The blue lines show the fit of a power law model fitted at the individual level; the orange lines show the joint fit of the mixed-effects model. The thin black lines are linear models. Note how overall the subjective level of discomfort tends to increase with MISC score; following a power law with an exponent $>1$. The dotted horizontal lines and associated terms show the median ME value for the verbal qualifiers participants attributed to particular ME. Individual panels are scaled by the maximum ME response given, which varies between individuals. Because ME ratings have no absolute meaning and vary between individuals, units are omitted on the *y*-axes. Note, however, that all lines cross the point MISC = 4, ME = 100 as per task instructions

**Fig. 4:**
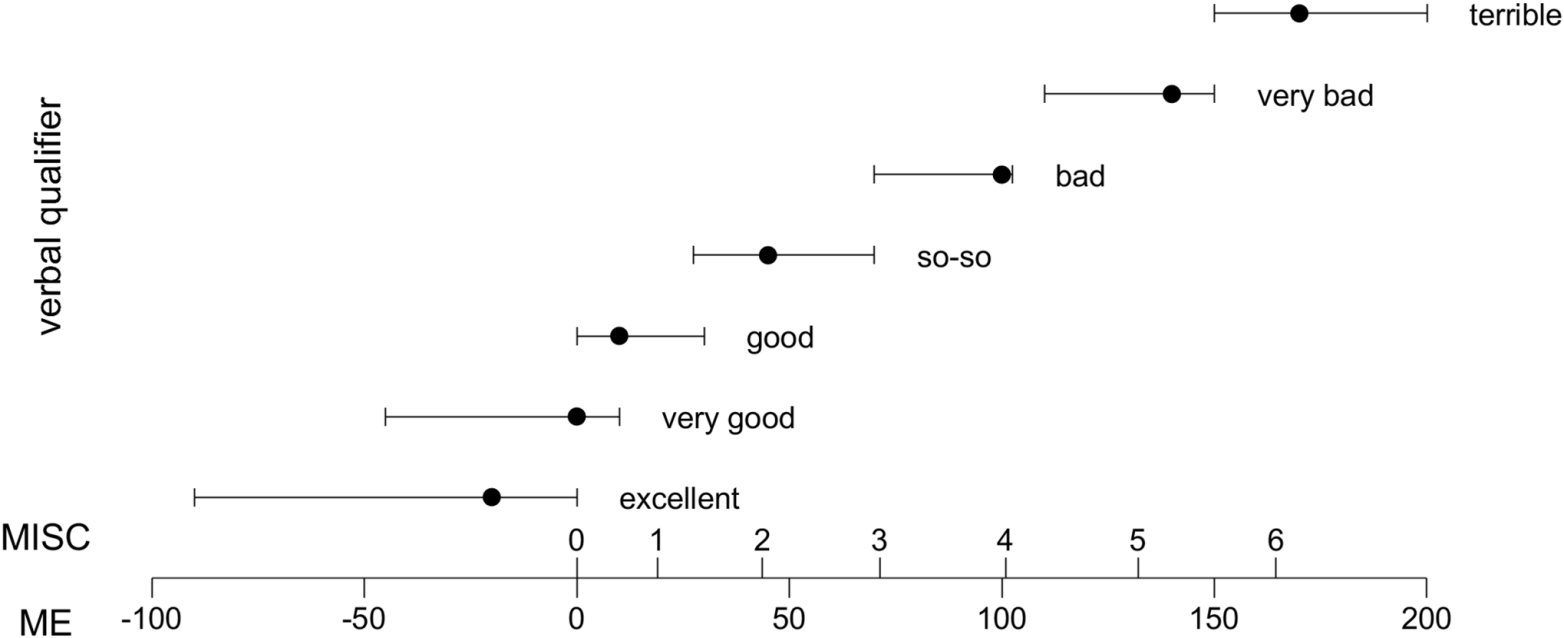
A visualization of the relation between MISC data, ME data, and verbal qualifiers collected in Experiment 2. The lower part of the figure shows the location of MISC scores on the ME scale according to the fitted model $\text {ME} = 18.945\times \text {MISC}^{1.206}$. The upper part shows the median (dot) and 25–75th percentiles (error bar) of ME scores associated with a series of verbal qualifiers. The figure shows that individuals tend to agree on feeling ‘terrible’ at an MISC of 6; feeling ‘so–so’ occurs between MISC scores of 1–3, and individuals differ with respect to the baseline level of comfort experienced, such that people tend to start out feeling ‘good’, rather than ‘excellent’. It can also be seen how the distance between MISC scores in terms of discomfort gradually increases between subsequent MISC scores. Note that (1) negative ME responses were allowed to account for cases where an individual may, for instance, not have experienced feeling ‘excellent’ at any time, and (2) no MISC data beyond ‘6’ were obtained, as the experiment was aborted as soon as a participant reached this score

The raw data and fit of the non-linear mixed-effects model are shown in Fig. 3 (orange lines), along with individually fitted linear (black lines) and power law models (blue). Model fit criteria for the jointly fitted linear and power law models were $AIC=4054.2/BIC=4062.0$ and $AIC=4047.7/BIC=4055.5$, respectively. This indicates that a power law is preferred. For the individually fitted linear and power law models, these values were $AIC=3299.0/BIC=3361.4$ (linear models) and $AIC=3169.4/BIC=3294.1$ (power law models), again indicating that a power law is preferred and also showing that there are considerable interpersonal differences in parameter values. Similarly, the mixed-effect models indicated a preference for a power law model ($AIC=3777.4/BIC=3781.3$) over a linear model ($AIC=3937.9/BIC=3940.1$), but the mixed-effect models did not account for the data as accurately as the individual fits. The latter finding implies that assumptions of the mixed-effect models (i.e., normally distributed random effects) did not match the data well, which is likely due to parameters for participants 3 ($a=3.885, b=2.276$) and 11 ($a=1.523, b=3.345$), which were outliers. Overall, the individually fitted power law models account for the data best. The median estimated values for coefficients *a* and *b* were 18.945 (25–75th percentiles: 12.777–24.546) and 1.206 (25–75th percentiles: 1.043–1.448). To interpret the subjective level of discomfort associated with ME, and to normalize these ratings between individuals, we asked participants to attribute ME as they used them during the experimental sessions to a set of verbal qualifiers. The dotted lines in Fig. 3 mark the locations of these judgments. The verbal qualifiers indicate that individuals tend to agree on feeling ‘terrible’ at a MISC of 6; feeling ‘so–so’ occurs between MISC scores of 1 and 3, and individuals differ with respect to the baseline level of comfort experienced, such that people tend to start out feeling ‘good’, rather than ‘excellent’ (see also Fig. 4). Note that when considering the scaling according to the verbal qualifiers, the data for the participants 3 and 11 agreed with the others, indicating that the differences in parameter estimates reflected idiosyncrasies in the numerical scaling for the ME task, but not so much in the verbal discomfort associated with MISC scores (as illustrated by the panels for these participants in Fig. 3).

A joint visual summary of the MISC data, ME data, and verbal qualifiers is given in Fig. 4. The lower part of the figure shows ME on a scale with evenly spaced points, and marks the location of associated MISC scores. The upper part shows the median (dot) and 25–75th percentiles (error bar) of ME/MISC scores associated with each verbal qualifier. The figure shows that both numerical and verbal subjective discomforts increase monotonously with MISC score.

## Discussion

We evaluated the relation between motion sickness and discomfort using a popular rating scale for direct assessment of an individual’s level of motion sickness; the MISC (Bos et al. 2005). The MISC orders symptoms according to a consensus of increasing severity and associates the symptoms with discrete scores, forming an ordinal scale (Stevens 1946). Because the ordering and clustering of symptoms that make up the scale can be disputed, and because the distance between successive points on an ordinal scale is not specified, analysis of this type of data may require non-standard analysis techniques, and it cannot be assumed that discomfort increases linearly with the MISC. The purpose of the present study was to determine whether these issues affect use of the MISC in practice. Specifically, we investigated (1) whether discomfort increases monotonously with MISC score and (2) how the relation between MISC and discomfort can be characterized. We found that discomfort indeed increases monotonously with MISC score, and that this relation is well captured by a power law with an exponent of 1.206. In the following, we discuss the findings in relation to the literature and consider their implications.

One particular concern regarding the MISC is that it probes multiple clusters of symptoms simultaneously. These clusters may, however, exist in parallel and have their own time courses. In addition, the ordering of the MISC may not completely match subjective reports on symptom progression (Bos et al. 2005). Consequently, it might be misleading to interpret the MISC as an indicator of the level of discomfort per se. This was recently illustrated by findings suggesting that the subjective level of discomfort drops between MISC scores of 5 and 6, reported by Reuten et al. (2020, 2021a, b). Findings from the semantic experiment confirm this observation. It has been suggested that this drop in the subjective level of discomfort between MISC 5 and 6 corresponds to an interval of relief of discomfort at the onset of nausea (Reuten et al. 2021a, b). Although similar observations have been reported previously (Reason and Graybiel 1969; Lackner 2014), we would expect nausea to develop *in the presence* of the other symptoms, and see no physiological reason for other symptoms to disappear at the onset of nausea. Indeed, when individuals are asked to report symptoms at the end of a motion exposure using the Motion Sickness Assessment Questionnaire (MSAQ; Gianaros et al. 2001), they typically report nausea along with all symptoms preceding nausea. Consequently, the conclusion that there is an interval of relief at the onset of nausea is counter-intuitive, and we believe that it is an artefact resulting from two methodological choices:

First, Reuten et al. (2020, 2021a, b) compared the progression of discomfort (unpleasantness) ratings obtained using the Fast Motion Sickness scale (FMS; Keshavarz and Hecht 2011) with MISC scores by evaluating the frequency and uniformity of the distribution of decreases among transitions between consecutive ratings. However, as the distributions of well-being data and motion sickness data were obtained from separate experiments, it cannot be ascertained whether any discrepancies between the distributions are indeed due to a non-monotonic relation between well-being and motion sickness, or due to fluctuations in the level of motion sickness (and, by association, well-being) over time. Moreover, the analysed FMS and MISC data were obtained from markedly different experimental paradigms, with FMS data obtained from participants exposed to constant velocity visual yaw-rotations (Nooij et al. 2017, 2021), and MISC data obtained from participants exposed to physical Off Vertical Axis Rotation (OVAR; Bos 2015) and 6-DoF motions (Bos et al. 2005). The comparison of the measurement methods is thus confounded by the variability in sickness due to the use of different stimulus modalities. This is problematic, because Visually Induced Motion Sickness (VIMS) is thought to be related to the experience of vection (i.e., visually induced sensations of self-motion), which can be an intermittent sensation (Nooij et al. 2017; Bonato et al. 2008; Kuiper et al. 2019), whereas the physical motions used to induce motion sickness provide continuous sensations of motion.

Second, the authors use various methods to rank the subjective valence of the items that make up the MISC: participants performed exhaustive pairwise comparisons (identical to our replication of that work in Experiment 1) and two variants of an ME paradigm, where participants drew lines with lengths corresponding to the discomfort associated with each item (Reuten et al. 2020, 2021a, b). As illustrated by the diverging findings of the two experiments reported here, an issue with ranking the subjective valence of the items of the MISC is that it is a semantic task, for which the findings may not correspond to how the scale is used in practice when rating sickness. In other words, in these paradigms, individuals may have judged the relative severity of the statements that make up the items of the MISC (which is a semantic task), rather than judging the subjective levels of overall motion sickness experienced during exposure to a motion stimulus (which is a perceptual task).

We replicated the finding from the pairwise comparison method used by Reuten et al. (2020, 2021a, b), showing a distinct drop in discomfort between MISC scores of 5 and 6. However, in a separate experiment, where we asked participants to associate MISC scores as reported under provocative conditions with discomfort ratings, we found that discomfort increases monotonously with MISC score.

It should be noted that in the experiment reported in the Reuten et al. (2021a, b) studies, participants also performed a task where they marked along a visual line (Visual Analogue Scale, or VAS) how bad they had felt during exposure to a motion stimulus, with the extremes of the lines corresponding to ‘very unpleasant’ and ‘very pleasant’. The latter ratings were plotted against the MISC scores obtained from the same participants. This method appears comparable to the method used in the present study, and therefore, similar results may be expected. In contrast to our findings, the mean VAS data did again suggest a drop between MISC scores 5 and 6. Even so, the drop was markedly smaller than for the verbal paradigms and was not statistically significant. Also, it should be noted that the presented data are based on single VAS scores obtained from individuals after a motion exposure. Consequently, there are no reference values for individuals, and therefore, it remains unknown whether an individual who, for instance, rates a MISC 6 as ‘mildly unpleasant’ would in fact rate MISC 5 as more unpleasant. Here, it should be noted that inferences of individual behaviour based on group averages may be misleading (i.e., the Ecological Fallacy). In contrast, we obtained corresponding ME for all MISC scores experienced for each individual.

A potential limitation of the present study is that associations between MISC and ME were made using a response sheet similar to Table 1; with the MISC items in order of appearance on the scale. As argued in the Methods section, given that participants used the MISC throughout the experiment, it is likely that they knew or recognized the order of individual items. Therefore, we chose not to apply a randomization of item order nor to omit the numbering for this task. It is possible that this affected the responses, favoring a hierarchical response on the ME as well. However, the verbal qualifier data were found to be consistent with the observed hierarchical association between MISC and ME, despite the fact that the items of this task were indeed presented in a random order, with additional instructions emphasizing that any scaling was allowed.

Taken together, we believe that there is a dissociation between how people interpret the statements that comprise the items of the MISC when asked to compare them explicitly and the use of the scale in practice in experiments where participants are generally made progressively more sick. This may have consequences for the interpretation of MISC data: for a strict use of the scale, study participants may need to be extensively trained before they are able to intuitively recognize individual symptoms and to attribute a certain number as a classifier to the particular symptom. In this case, it would also be necessary to analyse data with methods designed to deal with ordinal data. A parallel may be drawn to the use of the Cooper–Harper rating scale for aircraft handling qualities (Cooper and Harper 1969). Proper use of the scale requires a thorough understanding of the properties associated with each score, and confusion and misuse of the scale occur even among trained test pilots (Mitchell 2019).

When no explicit training in the use of the MISC is performed, MISC data may be best interpreted as ME, corresponding to an overall subjective level of motion sickness. Such use of a rating scale is intuitive, as a very similar method is used for nociception (pain perception) in medical settings without any training (e.g., Haefeli and Elfering 2006), and is consistent with its interpretation in the majority of studies reported in the literature. Used in this way, the MISC would be essentially equivalent to any scale ranging between “no-symptoms” and “vomiting”, such as the 11-point Well-Being scale (Reason and Graybiel 1969) or the 21-point FMS scale (Keshavarz and Hecht 2011), and a choice among them is arbitrary. The MISC has added value in that it may identify the presence of specific symptoms, but only when experimenters ascertain that participants are trained in proper use of the scale.

We found the subjective level of discomfort as function of MISC to be well captured by a power law up to MISC 6. We stopped our experiment when an MISC score of 6 was reached to increase the chances people would return for subsequent sessions, and subsequently have no data on the relation between higher levels of MISC and subjective levels of illness. Assuming, however, that there is an upper limit to subjective discomfort (culminating in vomiting), a sigmoidal relation may ultimately prove more appropriate. These findings should be considered when MISC data are used to fit mathematical models (e.g., Oman 1990) of motion sickness development, as the continuum on which predictions are made is often discomfort related, rather than on classification of specific symptoms.
